# Improving Cell Detection and Tracking in Microscopy Images Using YOLO and an Enhanced DeepSORT Algorithm

**DOI:** 10.3390/s25144361

**Published:** 2025-07-12

**Authors:** Mokhaled N. A. Al-Hamadani, Richard Poroszlay, Gabor Szeman-Nagy, Andras Hajdu, Stathis Hadjidemetriou, Luca Ferrarini, Balazs Harangi

**Affiliations:** 1 Department of Data Science and Visualization, Faculty of Informatics, University of Debrecen, 4032 Debrecen, Hungary; richardporoszlay@gmail.com (R.P.); hajdu.andras@inf.unideb.hu (A.H.); harangi.balazs@inf.unideb.hu (B.H.); 2 Doctoral School of Informatics, University of Debrecen, 4032 Debrecen, Hungary; 3 Department of Network and Computer Software Technologies, Hawija Technical Institute, Northern Technical University, Kirkuk 36001, Iraq; 4 Department of Microbial Biotechnology and Cell Biology, University of Debrecen, Life Sciences Building 1.102, 1 Egyetem Square, 4010 Debrecen, Hungary; szeman-nagy.gabor@science.unideb.hu; 5 Department of Information Technologies, University of Limassol, 3-5 Chaidariou Street, Limassol 3020, Cyprus; stathis11@gmail.com; 6 Department of Innovation Technologies, University of Limassol, 3-5 Chaidariou Street, Limassol 3020, Cyprus; luca@uol.ac.cy

**Keywords:** microscopy images, cell detection, YOLO model, cell tracking, DeepSORT algorithm, UKF, multi-scale ResNet50

## Abstract

Accurate and automated detection and tracking of cells in microscopy images is a persistent challenge in biotechnology and biomedical research. Effective detection and tracking are crucial for understanding biological processes and extracting meaningful data for subsequent simulations. In this study, we present an integrated pipeline that leverages a fine-tuned YOLOv8x model for detecting cells and cell divisions across microscopy image series. While YOLOv8x exhibits strong detection capabilities, it occasionally misses certain cells, leading to gaps in data. To mitigate this, we incorporate the DeepSORT tracking algorithm, which enhances data association and reduces the cells’ identity (ID) switches by utilizing a pre-trained convolutional network for robust multi-object tracking. This combination ensures continuous detection and compensates for missed detections, thereby improving overall recall. Our approach achieves a recall of 93.21% with the enhanced DeepSORT algorithm, compared to the 53.47% recall obtained by the original YOLOv8x model. The proposed pipeline effectively extracts detailed information from structured image datasets, providing a reliable approximation of cellular processes in culture environments.

## 1. Introduction

The convergence of biomedical research and artificial intelligence has catalyzed transformative advances in cellular analysis, with particular emphasis on automating microscopy-based workflows. This intersection has fostered technological innovations aimed at enhancing operational efficiency and analytical precision in biomedical applications. As the resolution and throughput of biological imaging systems continue to improve, the traditional manual analysis of cellular behaviors, such as detection, tracking, and division analysis, has become increasingly unscalable. In response, machine learning and computer vision have emerged as essential tools for scalable, high-fidelity analysis of dynamic cellular systems.

Among the most critical applications is the monitoring of cell life cycles, including proliferation, division, and morphological changes, which are essential for understanding disease progression, drug response, and developmental biology. Despite significant advancements in object detection, tracking, and segmentation, many existing models face challenges in the adaptability of models to diverse environments and the visibility of data in complex biological imagery [1,2,3]. These limitations highlight the need for more sophisticated and adaptable techniques to address a range of biotechnological applications, such as automated cell detection and tracking, which is the primary focus of this work.

Numerous advanced techniques and research studies have sought to address these challenges, significantly contributing to progress in this field. Among the most impactful advances is the You Only Look Once (YOLO) family of models, which has transformed the field of real-time object detection. In recent years, deep learning models have gained significant attention, particularly for solving detection problems. YOLO is a state-of-the-art single-stage detection model that performs object detection by analyzing the entire image in a single pass to identify objects and their locations [4]. This model was developed by Ultralytics and released in 2023. YOLO employs a convolutional neural network to predict bounding boxes and class probabilities. This streamlined approach contrasts with traditional techniques like R-CNN, which rely on more complex pipelines [5]. The YOLO series, comprising over ten versions, has revolutionized real-time object detection. Among these, YOLOv5, YOLOv8, and YOLOv11 are particularly notable for their ideal balance of speed, accuracy, and efficiency, making them well-suited for resource-limited scenarios, such as edge deployments [6]. For instance, Wu et al. [1] introduced a hybrid detection model combining the Swin Transformer and YOLO model to detect mycelium clamp connections and hyphae autolysis. This model demonstrated high efficiency, achieving an 89.02% mean Average Precision (mAP) at 31 FPS with a computational load of 98.6 GFLOPs. Similarly, Zou et al. [7] employed deep learning with Mask R-CNN to analyze bacterial communities in soil via microfluidic soil chips. These analyses, validated across diverse ecological samples, demonstrated robust detection capabilities, with (mAP, recall) pairs of (90%, 94%), (91%, 95%), and (89%, 96%) for Greenland, Sweden, and Kenya, respectively. Further, Haja et al. [2] developed a fully automated pipeline that employs a pre-trained Mask-RCNN model to segment yeast cell images, along with a YOLOv4 model to detect and classify individual cell compartments dividing the images into four quadrants. This method achieved significant time savings (up to 160 min), a 2–3% improvement in mAP, and a reduction in loss error.

Deep learning-based detection architectures, especially those based on the YOLO family, have achieved significant success in biomedical imaging. Wu et al. [8] improved YOLOv5 framework by incorporating the Swin Transformer into the backbone and refining the PANet architecture, achieving a mAP of 96% at 43.4 fps. Aldughayfiq et al. [9] combined a modified YOLOv5 with a Feature Pyramid Network in the field of cell counting in fluorescent microscopic images. The model uses a modified backbone of CSPs paired with a Feature Pyramid Network in the neck of the model to achieve significant improvements in cell counting, namely a detection precision rate of 91%, a mAP of 79.90%, and an average processing time of 43.9 ms over images of different sizes. Lopez et al. [10] suggested the use of a YOLOv5 model in the detection and enumeration of cells under fluorescent microscopic images. The model effectively detects and counts cells from images taken by the recommended CytoSMART Exact FL microscope. The model recorded an accuracy of 92%, a precision of 84%, a recall value of 91%, and an F1 score of 87% while maintaining a speed of 35.86 fps. Wahid et al. [11] discussed the most prominent challenges facing the implementation of yeast-based biosensors. The authors discussed problems such as shelf life, portability, the biological growth of certain yeast strands, and other technical difficulties. Solutions regarding the matters discussed are provided. Li et al. [12] introduced an improved YOLOv3 model to detect *Saccharomyces cerevisiae* infections from microscopic cell images. The proposed model implements the GhostNet in the backbone, as well as the HardSwish activation function and Complete Union over Intersection (CIoU) loss function. Compared to other methods, this model demonstrated superior performance in detecting *Saccharomyces cerevisiae* infections, achieving a 97.83% average precision and a detection time of 0.021 s for a single image. Zhang et al. [13] proposed a method for segmenting and detecting yeast cells in bright-field microscopy. The effectiveness of smooth spline and seeded watershed segmentation proved to be satisfyingly accurate and on par with manual segmentation and detection done by experts, with an approximate accuracy of 85–88%. Finally, Huang et al. [3] studied the impact of Fuzzy Automatic Contrast Enhancement on the performance of the YOLOv5 model for yeast cells detection. Their image enhancement approach improved the accuracy and confidence of the automated detection, resulting in an average accuracy of 94.20%.

Recent advancements in specialized object detection underscore the importance of contextual adaptation in both detection frameworks and the design of datasets. Zunair et al. presented RSUD20K [14], a comprehensive benchmark for road scene understanding that includes more than 20,000 images and 130,000 annotated bounding boxes. While RSUD20K 94 targets the autonomous driving domain, its emphasis on task-specific annotation protocols, cross-condition robustness, and model benchmarking-including YOLOv6, YOLOv8, and Transformer-based models-offers valuable methodological insights that can be applied in broader contexts. The study clearly illustrates that general-purpose models exhibit restricted transferability in the absence of domain adaptation—an analogous challenge is observed in biomedical imaging, where the complexity of microscopy-specific features necessitates meticulous fine-tuning. The findings highlight the significant importance of dataset-driven model refinement, which we implement in customizing YOLOv8x for microscopy-based cell detection.

**Figure 1:**
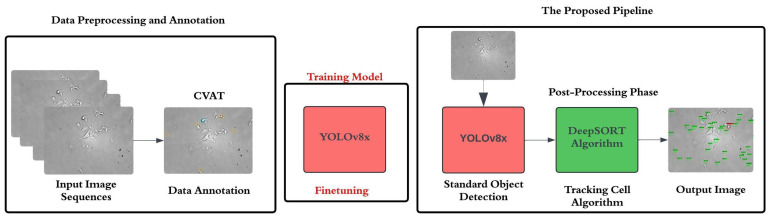
Flowchart of the integrated pipeline for cell detection and tracking using YOLOv8x and the DeepSORT algorithm.

Although these techniques achieve competitive results, they face several limitations, such as assumptions of linear object motion, the use of shallow appearance embeddings, and poor generalization to complex microscopy sequences. To address these challenges, we propose an integrated pipeline for automated cell detection and tracking that combines the high-speed object detection capabilities of YOLOv8x [15,16] with a customized version of the DeepSORT tracking algorithm [17,18]. The manuscript focuses on the automated analysis of microscopy data acquired from a real-time, long-term scanning imaging platform. The imaging setup has been developed at the University of Debrecen, functioning as a biological sensing system, as it is capable of continuously capturing cellular dynamics over time under near-infrared illumination. This integrated framework is used over the yeast microscopy dataset that consists of images labeled into two classes: cells and divisions. Our approach introduces significant improvements in both motion modeling and appearance representation, enabling more robust temporal association of cells under challenging visual conditions, as shown in Figure 1.

The main contributions of this work are as follows:

1. The integration of YOLOv8x with a modified DeepSORT module that replaces the conventional Kalman Filter with an Unscented Kalman Filter (UKF) for modeling non-linear cell motion;
2. The development of a multi-scale ResNet50-based visual descriptor to improve appearance-based data association and reduce identity switches;
3. The execution of a detailed ablation study to show the individual contributions of detection and tracking components on a microscopy dataset;
4. The demonstration of high-performance results on the microscopy dataset with incomplete annotations using Modified Recall and Average Intersection over Union (IoU).

The remainder of this paper is structured as follows: Section 2 details the materials and methods, including dataset characteristics, annotation procedures, and model architecture. Section 3 presents the experimental results and quantitative evaluations. Section 4 provides an in-depth discussion of the findings and their implications. Finally, Section 5 concludes the study and summarizes key contributions and potential directions for future research.

## 2. Materials and Methods

In vitro studies on cell and tissue cultures play an essential role in modern life sciences, offering essential insights into drug discovery, toxicological characterization, and biological interactions. Cell cultures are indispensable for understanding cancer biology, testing new compounds, and researching therapies. At the University of Debrecen, the Department of Microbial Biotechnology and Cell Biology has developed a non-invasive, near-infrared long-term scanning microscopy platform [19,20,21,22]. This approach eliminates illumination-induced experimental artifacts and enables cell examination at a temporal resolution of seconds for durations spanning several weeks. The high transmission of the 940 nm wavelength facilitates the monitoring of multilayered tumor development up to millimeters thick. An integrated perfusion system allows for seamless media exchange and manual dosing of active substances without interrupting the experiment. This platform or approach serves the basis for the microscopy dataset used in this study.

### 2.1. Dataset and Annotation Protocol

**Figure 2:**
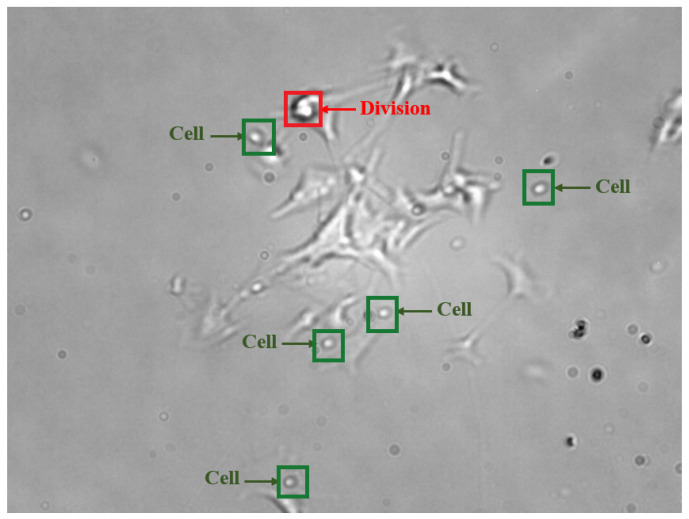
Sample frame from the B16BL6 dataset, where green labels indicate the Cell class, and the red label indicates the Division class.

The B16BL6 dataset used in this study comprises 1800 grayscale microscopy frames (1600 × 1200), captured from a continuous time-lapse video of dynamic cell activity. This dataset was provided by the Department of Microbial Biotechnology and Cell Biology at the University of Debrecen. Each frame visualizes cellular or subcellular structures with varying intensities of gray, as illustrated in Figure 2. To enhance the diversity of training samples, all frames were duplicated with an intensity transformation that inverted the grayscale levels, producing a darker variant of each original frame. This contrast-based augmentation strategy effectively doubled the dataset to 3600 frames, allowing the YOLO model to learn from diverse image variations, improving its ability to generalize and detect features across different intensity distributions and contrast levels.

In addition to grayscale inversion, we investigated conventional augmentation methods such as horizontal and vertical flipping, mild rotation, and contrast modifications. However, dealing with microscopy image datasets has a sensitive nature; for this reason, we deliberately refrained from applying aggressive geometric or color transformations that may compromise physiologically relevant structures. Although further augmentation, such as synthetic image generation, may enhance model generalization, it also carries the risk of overfitting to non-biological patterns. Therefore, we chose biologically plausible augmentations to preserve model integrity and interpretability. This strategy was designed in collaboration with domain experts to optimize performance improvements while mitigating the risk of overfitting.

Manual annotation was performed using the Computer Vision Annotation Tool (CVAT) [23], as shown in Figure 1. Ground truth labels were assigned to two biologically significant classes: the Cell class, which includes mitotically active cells in motion, and the Division class, comprising cells undergoing prophase or metaphase, often characterized by morphological changes such as increased area prior to cytokinesis. The annotation process was performed by a trained expert and verified by two domain specialists affiliated with the Department of Microbial Biotechnology. Discrepancies were adjudicated through consensus, ensuring high annotation fidelity.

The dataset exhibits a substantial class imbalance, with 1199 instances of the Cell class and only 39 instances of the Division class in the validation set. This pronounced disparity poses a challenge for training models to reliably detect less frequent, yet biologically important, Division events. To address this, we applied targeted data augmentation during training to mitigate this issue. This adjustment ensured balanced learning and reinforced the relevance of tracking in recovering rare Division events. To reduce the manual effort required for annotating all 1800 frames, a carefully selected subset was used for annotation, comprising 204 frames for training and 138 frames for validation. The selected frames focused on sequences starting during anaphase and telophase and ending when cytokinesis was complete, with two daughter cells visibly appearing. This selective approach ensured efficient annotation and minimized the need for manually annotating the entire dataset while maintaining the quality and representativeness of the dataset for training.

Although the Division class was defined by the morphological cues observed in prophase and metaphase, the annotated frame sequences were curated to capture the entire mitotic process, including anaphase, telophase, and cytokinesis. This methodological approach facilitates the model’s capacity to acquire knowledge not only of the appearance of dividing cells, but also their temporal evolution across biologically meaningful stages. Stationary, nondividing adherent cells were deliberately excluded from annotation, as their lack of dynamic behavior provided minimal utility for motion-based tracking. Our emphasis on mitotically active phases reflects their critical importance in studying chromatin reorganization, cellular morphology, and division dynamics-factors integral to cancer biology, developmental processes, and drug response assessment.

Each frame in our dataset contains between 10 and 22 annotated objects, reflecting a moderately sparse cellular environment typical of early-stage adherent cultures. While the majority of cells are well-separated, with no significant full-cell overlaps observed, some frames exhibit partial proximity or alignment, especially close to mitotic phases or during migratory interactions—which may introduce modest occlusion effects. Structural consistency is maintained in terms of cell type since all cells belong to the same cell type, which is a mouse cell line derived from melanoma. The primary variation lies in cell size and morphology, where the Division class typically appears larger and rounder (especially during prophase and metaphase), while the Cell class exhibits a broader range of sizes and intensities.

### 2.2. YOLO Model

In this study, we employed YOLOv8x, a state-of-the-art object detection architecture, which consists of four primary components: input, backbone, neck, and head. The input module manages image preprocessing tasks such as data augmentation and image normalization. The backbone extracts image features using modules such as Convolutional-BatchNorm-SiLu (CBS), Spatial Pyramid Pooling Fast (SPPF), and C2f modules to capture spatial hierarchies effectively. The Neck utilizes a Feature Pyramid Network (FPN) and a Path Aggregation Network (PANet) to enhance the model’s ability to detect objects of various sizes. This structure significantly improves detection accuracy, particularly for images with intricate backgrounds and objects of varying dimensions. The neck component in YOLOv8x, which generates feature pyramids, plays a crucial role in enabling the model to generalize across objects of varying scales and sizes. This feature pyramid approach, implemented through PANet, makes YOLO particularly effective in handling complex and small-scale objects, such as yeast cells [3]. PANet-based models have shown superior performance on previously unseen data, further solidifying YOLO’s robustness in diverse and challenging detection scenarios. Finally, the head uses a Decoupled Head structure to separate classification and detection tasks. Additionally, the traditional Anchor-Based technique has been replaced with the Anchor-Free concept, streamlining the learning process and improving detection accuracy, particularly for small or irregularly shaped objects [24].

YOLOv8 was chosen above previous YOLO variations due to its architectural improvements and its performance characteristics. In contrast to YOLOv5 and YOLOv7, YOLOv8 incorporates an anchor-free detection paradigm and a decoupled head structure, both of which boost generalization and robustness, crucial characteristics in microscopy-based biological applications. Moreover, its integration in PyTorch (2.6.0+cu124 (CUDA 12.4 build)) provides significant versatility and customization ease, enabling efficient model adaptation and fine-tuning. The combined enhancements establish YOLOv8x as an ideal choice for high-resolution, real-time analysis in intricate cell detection and tracking endeavors.

### 2.3. The DeepSORT Algorithm

Tracking algorithms and models are essential for providing continuous and accurate monitoring of objects, making them indispensable in various real-world applications. Relying on YOLO’s framewise detection fails to address common realistic scenarios, such as object occlusion, changes in appearance, and objects entering or leaving the field of view (FOV). Therefore, integrating object framewise detection with temporal tracking is necessary to analyze and gain insights, particularly in images and videos in microscopy, which contain multiple objects with varying trajectories [25].

**Figure 3:**
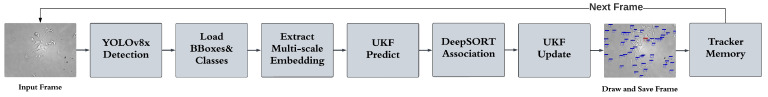
Enhanced DeepSORT Pipeline for Cell Tracking.

DeepSORT is an advanced multi-object tracking (MOT) algorithm that builds upon its predecessor, the Simple Online and Realtime Tracking (SORT) algorithm, by incorporating deep learning through a pre-trained convolutional neural network (CNN). This integration significantly enhances tracking performance, particularly in challenging scenarios such as occlusions [26]. DeepSORT processes detection outputs of the YOLOv8x as a postprocessing step, as shown in Figure 3.

The DeepSORT algorithm consists of three primary steps:

1. Target State Estimation

DeepSORT maintains a state representation for each tracked object using an 8-dimensional vector that captures both spatial properties and their first-order dynamics (velocities). The state vector is defined as

(1) $$x_{t}\;=\;\left[\begin{array}{c} x_{t}\\ y_{t}\\ a_{t}\\ h_{t}\\ v_{x,t}\\ v_{y,t}\\ v_{a,t}\\ v_{h,t} \end{array}\right],\;z_{t}\;=\;\left[\begin{array}{c} x_{t}\\ y_{t}\\ a_{t}\\ h_{t} \end{array}\right],$$

where

- $x_{t},y_{t}$: center coordinates of the bounding box,
- $a_{t}$: aspect ratio,
- $h_{t}$: height of the bounding box, and
- $v_{x,t},v_{y,t},v_{a,t},v_{h,t}$: velocities associated with each spatial component.

This formulation enables the model to anticipate the future state of each object, even in instances of missed detection, by utilizing temporal consistency. The measurement vector $z_{t}$ encompasses solely the observable elements (position and shape), which are directly acquired from the detector.

To estimate and propagate these states over time, we employ an Unscented Kalman Filter (UKF) [27], which is more robust to the non-linear dynamics typically observed in biological motion. The motion and measurement models are

(2) $$\begin{array}{cc} x_{t+1} & =Fx_{t} \end{array}$$

(3) $$\begin{array}{cc} z_{t} & =Hx_{t} \end{array}$$

where:

(4) $$F=\left[\begin{array}{cc} I_{4} & \Delta t\;I_{4}\\ 0 & I_{4} \end{array}\right],\;H=\left[\begin{array}{cc} I_{4} & 0_{4\times 4} \end{array}\right]$$

Here, F represents the constant velocity model, and H is the observation matrix.

We initialize the covariances as

(5) $$P_{0}=500\;I_{8},\;Q=0.1\;I_{8},\;R=2.0\;I_{4}.$$

These matrices define the uncertainty of the initial state $P_{0}$, the noise from the process Q, and the noise from observation R.

2. Data Association

To integrate appearance and motion cues, DeepSORT constructs a cost matrix C of matching detection J to track i $\;C_{ij}\;$ combine a motion term $d_{\mathrm{motion}}^{2}$ (e.g., Mahalanobis distance under the UKF),

(6) $$\begin{array}{cc} d_{\cos}\left(i,j\right)\; & =1-\frac{{\hat{e}}^{\;i}\cdot {\hat{e}}^{\;j}}{∥{\hat{e}}^{\;i}{∥}_{2}\;{∥{\hat{e}}^{\;j}∥}_{2}}, \end{array}$$

and an appearance term $d_{\cos}$ (cosine distance of $\ell_{2}$-normalized embeddings),

(7) $$\begin{array}{cc} C_{ij}\; & =\lambda \;d_{\mathrm{motion}}^{2}\left(i,j\right)\;+\;\left(1-\lambda \right)\;d_{\cos}\left(i,j\right). \end{array}$$

The optimal assignment of detections to tracks is computed using the Hungarian algorithm [28]. $\Pi \in {\left\{0,1\right\}}^{N\times M}$ is then found by solving the linear sum assignment problem subject to one-to-one constraints:

(8) $$\min_{\Pi }\;\sum_{i=1}^{N}\sum_{j=1}^{M}\Pi_{ij}\;C_{ij}\;s.t.\;\Pi \;1\leq 1,\;1^{⊤}\Pi \leq 1$$

3. Matching Cascade

DeepSORT is set apart from the original SORT algorithm by prioritizing the association of new detections with existing tracks. Tracks are categorized based on their age (i.e., the number of frames since they were last detected). Recently detected tracks are given higher priority, increasing the likelihood of correct reassignment. This approach effectively reduces identity switches and improves robustness when handling occlusions.

For the purpose of this work, we enhanced DeepSORT by modifying Steps 1 and 2:

1. Enhanced Motion Estimation

We replaced the linear KF with the UKF as shown in Equations (2) and (3), providing more accurate state estimations in scenarios with non-linear complex object motions by employing an unscented transform for calculating the mean and covariance.

2. Enhanced Appearance Embedding

We employ a multi-scale feature extraction strategy using a modified ResNet50 architecture [26]. Features were extracted from multiple convolutional layers {conv2_block3_out,conv3_block4_out,conv4_block6_out}. Denoting the *i*th feature map by $F_{i}\in R^{H_{i}\times W_{i}\times C_{i}}$, we apply global average pooling:

(9) $$e_{i}=\frac{1}{H_{i}W_{i}}\sum_{u=1}^{H_{i}}\sum_{v=1}^{W_{i}}F_{i}\left(u,v,:\right),$$

The resulting pooled vectors are concatenated: $e=[e_{1};e_{2};e_{3}]$, and $\ell_{2}$-normalize:

(10) $$\hat{e}=\frac{\left[e_{1};e_{2};e_{3}\right]}{∥\left[e_{1};e_{2};e_{3}\right]{∥}_{2}}$$

This approach improves the model’s ability to differentiate objects with similar appearances despite varying sizes or contextual backgrounds, thereby reducing identity switches and improving tracking continuity. The enhancements render the methodology particularly effective for precise cell tracking within complex biological systems.

## 3. Experiments and Results

The experiment was conducted utilizing the B16BL6 cell dataset in both its original and inverted forms. The inverted dataset comprised 3600 images; however, only a subset of these images was selected for training and validation as mentioned in Section 2.1. We used the extra-large YOLOv8 model (YOLOv8x). We trained the model using the default hyperparameter settings, and we experimented with different hyperparameters to enhance performance. We applied cosine learning rate scheduling (*cos_lr=True*), which adaptively adjusted the learning rate throughout training. Additionally, early stopping was implemented with a patience threshold of 5 epochs to prevent overfitting and optimize training efficiency. This combination of selective data subsets and tailored training parameters allowed for effective model fine-tuning while reducing the annotation overhead. Finally, the DeepSORT algorithm was applied to the original B16BL6 frame sequences to enhance detection performance.

The entire experiment was conducted in a Google Colab Pro environment, utilizing an NVIDIA Tesla T4 GPU with 15 GB of memory. The high-RAM runtime configuration was activated, providing access to approximately 25 GB of system memory. CUDA acceleration was employed during both the training and validation phases of the YOLOv8x model, as well as during the execution of the enhanced DeepSORT algorithm. All components were implemented in Python 3.11 to ensure modularity and scalability.

### 3.1. Evaluation Metrics

To evaluate the performance of our integrated pipeline, we focused on two primary metrics (Modified Recall and Average IoU), due to the incompleteness of our ground truth annotations. Using standard metrics such as Precision and mean mAP could mislead the overall evaluation because they penalize detections that are not present in the ground truth, even though these detections represent valid objects that were missed during annotation, as mentioned in Section 2.1. As a result, these additional detections would be classified as false positives (FPs), which would not accurately reflect the model’s actual performance. To overcome this limitation, we used Modified Recall and Average IoU, which provide a comprehensive assessment of detection and tracking performance.

Modified Recall focuses on the model’s ability to capture the annotated (ground truth) objects. By ignoring extra predictions (i.e., not penalizing false positives), this metric reflects the completeness of detection with respect to the annotated subset. Modified Recall is computed over the validation set as follows:

(11) $$\mathrm{Modified}\;\mathrm{Recall}=\frac{\sum_{\mathrm{images}}TP}{\sum_{\mathrm{images}}\left(TP+FN\right)}$$

where TP represents true positives, and FN represents false negatives.

At the same time, we used Average IoU to measure the overall localization quality of the detections, which precisely evaluates how well the predicted bounding boxes overlap with the ground truth. It is calculated as follows:

(12) $$\mathrm{Average}\;\mathrm{IoU}=\frac{\sum_{i=1}^{N}{\mathrm{IoU}}_{i}}{N}$$

where N is the total number of matched ground truth-prediction pairs.

### 3.2. Ablation Study

To evaluate the contribution of each pipeline component, we conducted an ablation study on the B16BL6 dataset using three configurations:

1. YOLOv8x (Original Model):
  We used the baseline model (standard pre-trained YOLOv8x) architecture with its default parameters. This step serves as a reference to assess the impact of the fine-tuned and tracking algorithm.
2. YOLOv8x (Fine-tuned Version):
  This model is fine-tuned YOLOv8x over the B16BL6 dataset by using Cosine learning rate scheduling and early stopping strategies to improve generalization and reduce the overfitting problem.
3. YOLOv8x (Fine-tuned) + Enhanced DeepSORT (UKF + ResNet50):
  The integrated pipeline of fine-tuned YOLOv8x and the enhanced DeepSORT tracking algorithm that incorporates the UKF for robust motion modeling and a multi-scale ResNet50-based appearance descriptor to reduce identity switches.

The model’s performance was evaluated on the validation set for both the original and the enhanced YOLOv8x model. The enhanced model demonstrated notable improvements in detection performance, particularly when cells exhibited well-defined boundaries, as shown in Table 1. However, the model faced some limitations in detecting occluded or overlapping cells. At the end of the detection phase, it was observed that the model remained prone to missing certain cell types between frames, some of which led to false negatives.

**Figure 4:**
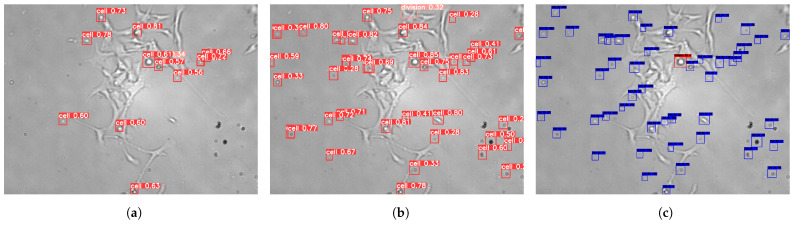
Qualitative comparison of detection and tracking outputs across the original YOLOv8x, fine-tuned YOLOv8x, and the proposed Enhanced DeepSORT pipeline. Enhanced DeepSORT significantly improves trajectory continuity and identity preservation, especially in situations involving overlapping or temporarily occluded cells. (**a**) Original YOLOv8x. (**b**) Fine-Tuned YOLOv8x. (**c**) Enhanced DeepSORT.

To address these issues, the enhanced DeepSORT algorithm was utilized. This algorithm identified missed detections by examining frames where cells were not detected, reviewing the annotations, and comparing them to the model’s predictions, as shown in Figure 4. Following the implementation of the integrated pipeline, the enhanced DeepSORT algorithm outperformed both the original and enhanced YOLOv8x models, as detailed in Table 1.

The results underscore the effectiveness of integrating the enhanced YOLOv8x with the enhanced DeepSORT algorithm, particularly for tracking and detecting cells that were initially occluded or undetected. This highlights the importance of robust tracking algorithms for improving overall detection accuracy and reducing false negatives. This internal comparison evaluates the contributions of each component in our pipeline, as no prior benchmarks exist for this newly introduced dataset. To further support these results, we have published frame sequence samples in [29], which clearly demonstrate the enhanced performance of our proposed solution in detecting and tracking cells.

To augment the aforementioned assessment, we evaluated execution time across configurations. The average time per frame for the YOLOv8x (Original Model) was 0.055 s, while the YOLOv8x (Fine-tuned Version) enhanced both performance and speed, achieving 0.047 s per frame. Our full integrated pipeline YOLOv8x (Fine-tuned) + Enhanced DeepSORT (UKF + ResNet50), incurred substantial computational overhead due to complex motion modeling and feature extraction, yielding an average processing time of 8.5 s per frame. These results reflect a trade-off between real-time inference and tracking robustness.

The process depicted in Figure 1 and Figure 3 demonstrates a sequential pipeline, commencing with YOLOv8 detection and subsequently employing DeepSORT tracking; nevertheless, the tracking module significantly enhances the overall practical recognition rate. The DeepSORT method, utilizing the Unscented Kalman Filter (UKF), predicts cell positions during transient detection failures or occlusions, therefore reducing persistent missed detections. Additionally, the multi-scale ResNet50 appearance embeddings enhance the accuracy of identity reassociations across frames, ensuring consistent cell trajectories and identities despite intermittent detection inaccuracies. The complementary interplay between detection and tracking improves recognition continuity and resilience during image sequence analysis, even though the workflow is sequential in structure.

#### Cross-Dataset Generalization on the VISEM-Tracking Dataset

To further validate the generalization capability of our proposed pipeline, we conducted a transfer assessment on the publicly available VISEM-Tracking dataset [30], which consists of 29,196 annotated frames of spermatozoa imaged captured by phase-contrast microscopy with three classes (normal sperm, pinhead, and cluster). We selected a continuous subset of 1500 frames to maintain consistency in evaluation scale with our original B16BL6 dataset. In contrast to our melanoma-derived mouse cell imagery, VISEM-Tracking exhibits a distinctly different biological context, featuring human sperm cells with rapid non-linear motion and varied morphological profiles.

We evaluated the selected subset of the VISEM dataset with the same three model configurations described previously:

Table 2 demonstrates that our full pipeline achieves near-perfect recall and a substantial enhancement in spatial alignment accuracy (Average IoU), indicating that the improvements introduced via enhanced motion modeling and appearance embeddings generalize well across distinct imaging modalities and cell types. These results indicate that our methodology is not confined to a single dataset but can be modified for many biological contexts where continuity, identity retention, and resilience to motion dynamics are essential.

### 3.3. Computational Complexity

To evaluate the computational complexity of our proposed architecture, we performed a detailed assessment of both the complexity of the model (parameter count) and the inference cost (GFLOPs per frame). This includes the original YOLOv8x model, the fine-tuned YOLOV8x version, and our integrated detection-tracking pipeline with the enhanced DeepSORT module.

The results are summarized in Table 3. Note that we follow the Ultralytics convention, counting each multiply and add as separate FLOPs (i.e., GFLOPsUltralytics = 2 × MACsTHOP). The reported 974.6 GFLOPs of the full pipeline encompass object detection and the DeepSORT tracking module, which includes a modified ResNet50 for embedding extraction across all active objects (averaging N = 57 per frame). This total also accounts for additional computational overhead introduced by non-linear motion modeling via the Unscented Kalman Filter (UKF), cosine similarity computations, and data association using the Hungarian algorithm. As expected, the inference cost scales approximately linearly with the number of active tracks.

Despite the increased computational burden from sophisticated motion modeling and multi-scale feature extraction, the marginal rise in parameters and operations is justified by the substantial improvements achieved in detection continuity, identity preservation, and overall tracking robustness.

## 4. Discussion

Our proposed pipeline, which is based on a deep learning framework, is applied directly to data collected by this sensor-based microscopy platform. The essential goal is to improve real-time cellular event detection and tracking, which is critical for phenotypic sensing, drug testing, and dynamic biological monitoring. The proposed integration of the YOLOv8x detector with the enhanced DeepSORT tracking algorithm effectively addresses key limitations in previous approaches to automated cell analysis. The improvement in the DeepSORT algorithm in terms of adding non-linear motion modeling and deep multi-scale appearance descriptors substantially improves temporal consistency for detection and tracking the cells, which lies at the core of this study.

Fine-tuning the YOLOv8x model using cosine learning rate scheduling and an early stopping patience threshold further enhanced its performance relative to the base model, helping prevent overfitting. Simultaneously, UKF integration within the DeepSORT framework enables robust modeling of non-linear cellular trajectories, while the multi-scale ResNet50 embeddings improve discriminability between visually similar cells across consecutive frames. These combined innovations yielded significant improvements in both recall and tracking stability.

Our integrated pipeline is modular and flexible, allowing for use across many cell types and imaging techniques. Domain-specific fine-tuning enables the re-optimization of detection and tracking components to address variations in cellular morphology, contrast, and motion dynamics.

However, this study presents a key limitation. The incomplete ground truth annotations limited our ability to compute comprehensive detection and tracking metrics such as mean Average Precision (mAP), IDF1, and MOTA.

Despite these limitations, the proposed framework demonstrates considerable promise for longitudinal cellular analysis. It provides a foundation for tracking mitotic behavior, monitoring cellular migration, and capturing phenotypic variability under diverse experimental conditions. Future work will focus on applying transformer-based models for temporal reasoning and enhancing the ground truth annotations by using semi-supervised learning techniques.

## 5. Conclusions

In this study, we developed an integrated pipeline that combines the well-known YOLOv8x model for the framewise detection phase with the DeepSORT algorithm to enhance the detection and continuous tracking of cells and cell divisions in microscopy images. By incorporating an Unscented Kalman Filter and multi-scale ResNet50 features, the proposed framework significantly improves detection continuity and tracking accuracy.

Our system achieved a Modified Recall of 93.21%, a substantial improvement over the 53.47% achieved by the original YOLOv8x model. This result affirms the efficacy of our approach for detailed, providing a general and powerful tool for extracting meaningful information from microscopy images, which is crucial for understanding biological mechanisms and supporting subsequent analyses, such as modeling cellular behaviors in diverse environments. Although our proposed pipeline achieved higher recall, future research with improved annotations should aim to re-assess Precision and mAP for a more comprehensive evaluation of performance.

## Figures and Tables

**Table 1:** Performance metrics measured on the B16BL6 dataset.

Model Configuration	Modified Recall	Average IoU	Detected Cells	Missed Cells	Time per Frame (s)
YOLOv8x (Original Model)	53.47%	77.82%	662	576	0.055
YOLOv8x (Fine-tuned Version)	85.95%	76.77%	1064	174	**0.047**
YOLOv8x (Fine-tuned) + Enhanced DeepSORT (UKF + ResNet50)	**93.21%**	76.73%	**1154**	**84**	8.5

**Table 2:** Generalization performance on the VISEM-Tracking dataset.

Model Configuration	Average IoU	Modified Recall
YOLOv8x (Original Model)	0.8349	99.4%
YOLOv8x (Fine-Tuned)	0.8179	99.0%
YOLOv8x (Fine-Tuned) + Enhanced DeepSORT (UKF + ResNet50)	**0.9623**	**99.9%**

**Table 3:** Model complexity metrics across detection and tracking configurations.

Model Configuration	Parameter Count	GFLOPs per Frame
YOLOv8x (Baseline)	68,229,648	258.5
YOLOv8x (Fine-Tuned)	68,154,534	258.1
YOLOv8x (Fine-Tuned) + Enhanced DeepSORT (UKF + ResNet50)	76,743,718	974.6

## Data Availability

The dataset used in this study is not publicly available.
